# Limiting the Spectral Diffusion of Nano-Scale Light Emitters using the Purcell effect in a Photonic-Confined Environment

**DOI:** 10.1038/s41598-018-37677-2

**Published:** 2019-02-04

**Authors:** A. Lyasota, C. Jarlov, A. Rudra, B. Dwir, E. Kapon

**Affiliations:** Laboratory of Physics of Nanostructures, Institute of Physics, Ecole Polytechnique Fédérale de Lausanne (EPFL), Lausanne, Switzerland

## Abstract

Partial suppression of the spectral diffusion of quantum dot (QD) excitons tuned to resonance of a nano-photonic cavity is reported. The suppression is caused by the Purcell enhancement of the QD-exciton recombination rate, which alters the rate of charging of the solid-state environment by the QD itself. The effect can be used to spectrally-stabilize solid-state emitters of single photons and other non-classical states of light.

## Introduction

Electric charging in the environment of nano-scale light emitters can lead to spectral diffusion or intermittency (blinking) of the emission, as observed with nitrogen-vacancy centers in diamond^1^, molecules^2^, carbon nanotubes^3^, semiconductor nanorods^4^ and quantum dots^5,6^ (QDs) embedded in solid-state matrices. Various aspects^7–11^ and mechanisms^9,12–14^ of such spectral dynamics were widely investigated for semiconductor QDs, partly in an attempt to quench these effects. In particular, engineering of colloidal QD environment^15,16^ and its nanostructure^17–19^ provided stable nano-emitters with suppressed emission intermittency useful for quantum science and technology applications. While Purcell enhancement of a QD radiation rate through coupling of a QD transition to a plasmonic or a hybrid mode^20–22^ strongly quenches non radiative processes including the ones that lead to emission intermittency, we did not find any report on an effect of modified photonic environment on the QD spectral diffusion. Here, we report on the suppression of the spectral diffusion of QD-excitons incorporated in photonic cavities. It is shown that, for QD emission tuned to the resonance of the cavity, the Purcell enhancement of the exciton radiative recombination leads to reduced electrical charging of the QD environment, which, in turn, reduces the spectral diffusion range. Moreover, the experiments and modeling evidence a QD environmental self-charging due to carriers trapped in the dot itself, supporting reports on similar QD charging leading to emission intermittency in open photonic environment^23^.

## Results

**Figure 1:**
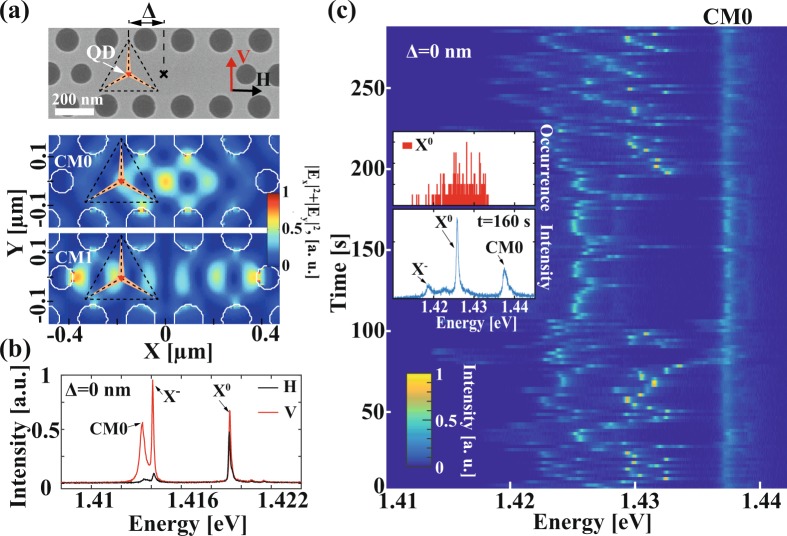
(**a**) SEM top view of the fabricated structure and computed near-field intensity patterns of modes CM0 and CM1. (**b**) Polarization-resolved photoluminescence (PL) spectra showing exciton-cavity mode weak coupling (T = 10 K). (**c**) Temporal-spectral map showing PL spectra acquired sequentially for a structure exhibiting “free” X spectral diffusion. Excitation power P_exc_ = 300 µW, T = 10 K. Insets show spectrum acquired at t = 160 s and occurrence histogram of the neutral excitonic transition X°.

Our photonic–confined QD system presented in Fig. 1(a) consists of a single InGaAs/GaAs site-controlled QD grown in an inverted pyramid, around which a photonic crystal (PhC) membrane cavity was defined (see Methods). We designed the QD emission wavelength to be close to resonance with the fundamental cavity mode CM0 and the first excited mode CM1 (Fig. 1(a)). Weak CM-QD coupling was verified by observing the co-polarization between the QD exciton (X) and the CM line for sufficiently small X-CM detuning^24^ (Fig. 1(b)). Such co-polarization is accompanied by reduced X lifetimes, both arising from the Purcell effect^25^. From the co-polarization parameters we estimate a Purcell factor of F_p_ ~ 10 for studied structures (see Methods).

Whereas most structures showed PL spectra stable over acquisition time, several samples exhibited spectral diffusion of the QD transitions (Fig. 1(c)). We attribute this spectral diffusion mainly to the fabrication-induced defects around the QDs, which can trap electric charges and induce spectral shifts via the quantum confined Stark effect and bi-exciton binding energy modifications^26^. It should be noticed that almost all QDs stopped diffusing spectrally if exposed to above-barrier excitation during several tens of minutes. We explain this effect by the quasi-stable states of defects or background impurities that are reached after a long exposure to photoexcitation and hence high densities of excited electron-hole pairs. For CM-X detuning of at least several meV, as in Fig. 1(c), we did not observe any effect of the CM on the spectral diffusion.

**Figure 2:**
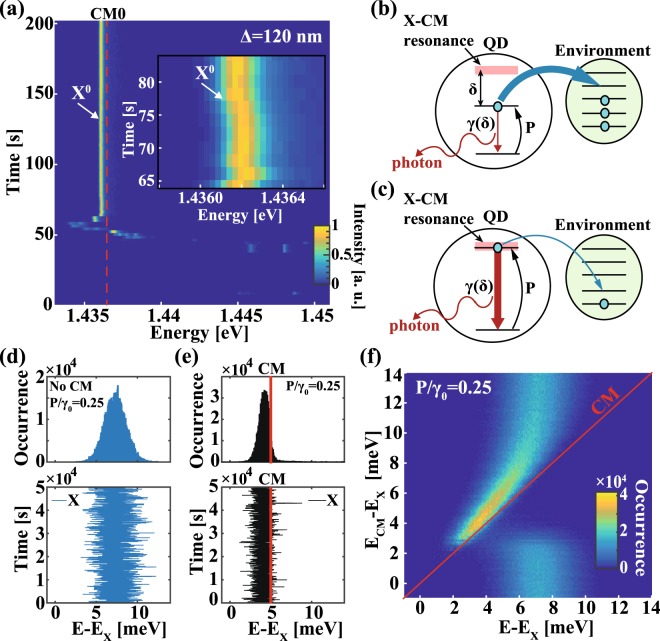
(**a**) Temporal-spectral map for an L3 PhC cavity with Δ = 120 nm showing spectral trapping of the X by the CM mode (red dashed line). Inset shows a detail of the spectrally diffusing exciton at t = 75 s. Excitation power P_exc_ = 100 µW, T = 10 K. (**b**,**c**) Schematics of the model showing the QD and charged environment for high (**b**) and low (**c**) charging rates, controlled by the X-CM detuning. Simulated X spectral diffusion: temporal trace and occurrence histogram without (**d**) and with (**e**) the CM. (**f**) Simulated map of the X occurrence versus relative CM energy E_CM_ − E_X_ for quantum dot population rate P = 0.25γ_0_ where E_X_ is unperturbed X energy (see Methods for details on simulations).

However, for sufficiently small CM-X detuning, we observed spectral diffusion within a restricted energy range, confined to the vicinity of the CM resonance (Fig. 2(a)). We explain this “spectral trapping” of the otherwise freely diffusing X transition by the combined effects of the enhanced X emission rate γ due to the CM-induced Purcell enhancement at sufficiently small X-CM detuning δ, and the role of the QD as a primary source of the charging for the traps in its vicinity (Fig. 2(b,c)). Similar charging mechanism leads to exciton lifetime-intensity correlations in emission intermittency of colloidal QDs^27^, recently also reported for InGaAs/GaAs QDs^23^. In this scenario, the charging rate of the surrounding traps is proportional to the QD average occupation II with an electron-hole pair. The latter is estimated as $\,{\rm{\Pi }}=\frac{1}{1+{\rm{\gamma }}(\delta )\,/\,{\rm{P}}}$ from the steady state solution of the rate equation $\frac{d{\rm{\Pi }}}{dt}={\rm{P}}(1-{\rm{\Pi }})-{\rm{\gamma }}({\rm{\delta }}){\rm{\Pi }}$ driving the single electron-hole dynamics in the QD at a population rate P below the X saturation level. The coupling of the X transition with the CM modifies the QD population depending on the X-CM detuning δ = E − E_CM_, where E and E_CM_ are exciton and CM energies. The Purcell effect on the X recombination rate reads^28–30^

1 $${\rm{\gamma }}({\rm{\delta }})=\frac{{{\rm{\gamma }}}_{0}{{\rm{F}}}_{{\rm{P}}}(1+\frac{{{\rm{Q}}}_{{\rm{CM}}}}{{{\rm{Q}}}_{{\rm{X}}}})}{8\frac{{{\rm{\delta }}}^{2}}{{{{\rm{\Delta }}{\rm{\omega }}}_{{\rm{CM}}}}^{2}}+2{(1+\frac{{{\rm{Q}}}_{{\rm{CM}}}}{{{\rm{Q}}}_{{\rm{X}}}})}^{2}}{{\rm{\eta }}}^{2}+{{\rm{\gamma }}}_{{\rm{leak}}}$$

where γ_0_ and γ_leak_ are the X spontaneous emission rates in the bulk (open photonic environment) and in the PhC gap, F_P_ is the theoretical Purcell factor, ${{\rm{Q}}}_{{\rm{CM}}}=\frac{{{\rm{\omega }}}_{{\rm{CM}}}}{{{\rm{\Delta }}{\rm{\omega }}}_{{\rm{CM}}}}$ and ${{\rm{Q}}}_{{\rm{X}}}=\frac{{{\rm{\omega }}}_{{\rm{X}}}}{{{\rm{\Delta }}{\rm{\omega }}}_{{\rm{X}}}}$ are the CM and the X quality factors defined through the X and the CM angular frequencies, and η is the effective CM-X spatial overlap^25^. Thus, the charging rate of the QD environment and the spectral diffusion of the excitonic transitions are coupled to the X-CM detuning δ = E − E_CM_ (Fig. 2(b,c)). It should be noted that before exciton X^0^ started spectrally diffusing (Fig. 2(a)), its emission was almost completely quenched^5,6^ during the first 36 s due to the high electric field induced by the environmental charging. This quenching is caused by either QD ionization or by a reduced dipole moment of the QD transition.

In this picture, the charging level of the surrounding traps is proportional to the QD average occupation with an electron-hole pair, which depends on the QD pumping rate P and the recombination rate γ of the QD-confined exciton. The QD recombination rate, in turn, increases with decreasing CM-X energy detuning δ. Thus, the QD environment is more efficiently charged when the QD optical transition is tuned out of resonance with the CM (Fig. 2(b)). On the other hand, at resonance the electron-hole recombination rate is enhanced by the cavity Purcell effect, which reduces the average QD occupation and consequently decreases the charging rate of the traps (Fig. 2(c)). At large CM-X detuning and high charging levels, the electric field, exerted by the environment, shifts the X-transition provoking spectral diffusion. For small CM-X detuning, the charging level of the environment can be drastically lowered, which reduces the X-level shifts and the consequent spectral diffusion. Here, we neglected the effect of carrier losses due to traps charging on the average QD occupation. This is because such loss rate is comparable to that of spectral diffusion, that is, a few Hz, and is at least 7–8 orders of magnitude smaller than the X recombination rate. The latter varies between 2 GHz to 20 GHz, as can be extracted from the ~2 ns and ~200 ps radiative-limited excitonic lifetime in an open photonic environment and in direct resonance with the cavity mode (δ = 0), respectively.

Figure 2(d),(e) show the simulated time traces and occurrence histograms of the X-transitions as a function of the energy shift from the unperturbed (i.e., without energy shift) X energy E_X_ (see Methods for details on the numerical model). Evidently, the presence of the cavity reduces the range of spectral diffusion of the X-line, pulling the recombination energy to the vicinity of the CM. At the same time, in the presence of the CM the occurrence histogram is suppressed at the CM energy, with its peak located at one side of the CM energy. A more complete description of the occurrence distribution is shown in Fig. 2(f), which displays the simulated spectrum of the occurrence as a function of the CM energy E_CM_. The occurrence assumes its maximum at either side of the CM, a manifestation of the Purcell-enhanced recombination of excitons near the cavity resonance.

**Figure 3:**
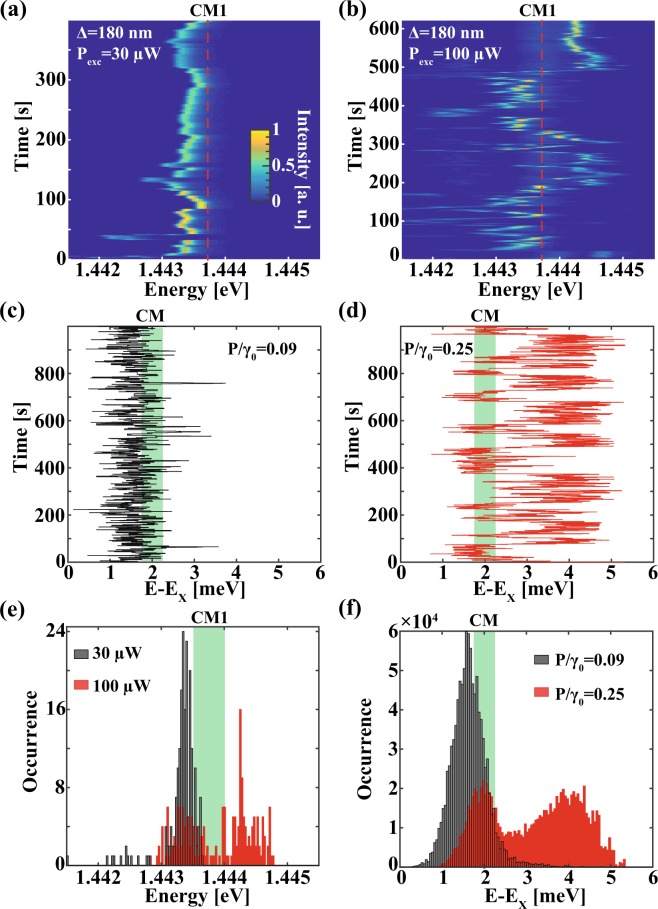
Measured (**a**,**b**) and simulated (**c**,**d**) excitonic trajectories for two different photo-excitation powers and QD pumping rates respectively. Corresponding measured and simulated excitonic occurrences are shown in (**e**) and (**f**). Experimental data in (**a**) and (**b**) were acquired at P_exc_ = 30 µW and P_exc_ = 100 µW excitation powers (QD in an L3 PhC cavity with Δ = 180 nm, mode CM1) and temperature T = 10 K. Simulated excitonic trajectories in (**c**) and (**d**) were obtained for quantum dot population rates P = 0.09γ_0_ and P = 0.25γ_0_ (See Methods for details on simulations). Same color scales used in (**a**) and (**b**).

Further support to our model is provided by the dependence of the spectral diffusion pattern on the photo-excitation power P_exc_ (Fig. 3(a,b)), measured in another device. Increasing the QD pumping rate shifts the peak occurrence of the X transition to higher energies and produces wider spectral diffusion range, reaching across the CM resonance. This is brought about by the higher average charging of the environment and resulting intense electric fields. Remarkably, the measured occurrence of the neutral exciton transition is depleted close to the CM energy, exhibiting a clear dip in the occurrence spectrum for larger excitation powers (Fig. 3(e)). Our numerical model reproduces this behavior, as reflected in both the simulated spectral diffusion traces (Fig. 3(c,d)) and spectral occurrence histograms (Fig. 3(f)). In these simulations we used 20 single-electron traps in the environment, with excitonic shifts uniformly distributed between E_min_ = −0.06 and E_max_ = 0.54 meV, and corresponding charging and discharging rates lower then f_max_ = 10 Hz and l_max_ = 0.1 Hz. It can be seen that the key parameter controlling the spectral diffusion pattern is the normalized pumping rate P/γ_0_.

Additional insight into the impact of the photonic environment on the spectral diffusion is obtained by a simple analytic model for the temporal evolution of the exciton energy *E*. Assuming that the energy shift *E* − E_*X*_ of the excitonic transition is proportional to the total charge accommodated in the QD environment *Q* (*E* − E_*X*_ ~ *Q*), we can write

2 $$\frac{dE}{dt}=\frac{P\alpha }{\gamma (E-{{\rm{E}}}_{CM})+P}({{\rm{E}}}_{max}-E)-\beta (E-{{\rm{E}}}_{X})$$

where E_*X*_ and E_*max*_ correspond to zero and maximum charge Q, *γ*(*E* − E_*CM*_) is the exciton emission rate, α and β are charging and discharging rates (see Methods for details of the analytic model). For simplicity, we ignore the stochastic nature of the charging process, thus the exciton energy converges to well-defined values at infinite time. Analysis of the equation permits the determination of “stable” exciton energies, which represent the energies of highest occurrence during the spectral diffusion process.

**Figure 4:**
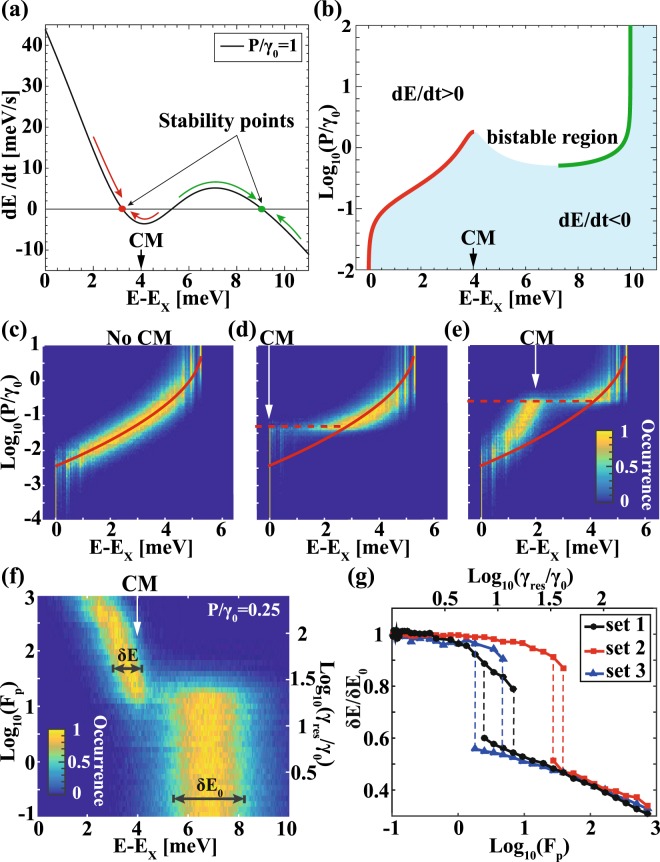
(**a**) Determination of the stability points in the exciton energy spectrum via the analytic model. (**b**) Calculated stability zones in the QD population rate-exciton energy plan. (**c**–**e**) Numerically simulated QD population rate- exciton energy maps of excitonic occurrence without and with CM at different energy. Solid red lines in (**c**–**e**) trace the energies of the occurrence peak without the CM. (**f**) Purcell factor-energy map of exciton occurrence and (**g**) normalized FWHM of exciton occurrence as a function of Purcell factor. Dashed red lines in (**d**) and (**e**) show the transition (normalized) power above which the occurrence peak energy recovers its values in (**c**). Dashed lines in (**g**) highlight ${{\rm{F}}}_{{\rm{p}}}\approx {{\rm{F}}}_{{\rm{p}}}^{{\rm{th}}}$ values at which exciton diffusion is only partially limited to the CM side.

Figure 4(a) displays the derivative $\frac{dE}{dt}$, calculated from (2), for typical parameters of our system (See Methods). The stationary points *E*_*stat*_ satisfying $\frac{{d}^{2}E({E}_{stat})}{d{t}^{2}} < 0\,$are indicated. In an intermediate range of QD population rate P, two points are obtained (Fig. 4(b)), such that the exciton transition most likely occurs above or below the CM energy. At higher QD population rates, a single stable branch exists, restoring the spectral diffusion to its pattern in the absence of a CM. As shown in Fig. 4(b), the energy at which spectral diffusion is captured depends on a particular detuning between unperturbed excitonic energy E_X_ and CM energy. We corroborated this result with numerical simulations as shown in Fig. 4(d,e). Figure 4(d,e) demonstrate that the spectral diffusion ‘capture’ energy, as well as the range of QD pumping rates at which QD spectral diffusion is limited near E_CM_, strongly depend on the E_X_ - E_CM_ detuning. The free spectral diffusion of the QD transition in the absence of a cavity (Fig. 4(c)) persists at higher QD pumping rates when the CM is tuned closer to the unperturbed excitonic energy E_X_ (Fig. 4(d)), and bunches near the CM energy for sufficiently low QD population rates (Fig. 4(e)). In all cases, the spectral diffusion in the absence of CM (Fig. 4(c)) is recovered above a threshold QD population rate, which depends on the E_CM_ - E_X_ value.

Figure 4(f) shows spectral diffusion ranges as a function of Purcell factor F_p_ and corresponding emission rate enhancement in resonance γ_res_/γ_0_ obtained using Eq. (1). Exciton occurrence is limited to the CM side for F_p_ above some threshold value ${{\rm{F}}}_{{\rm{p}}}^{{\rm{th}}}$ with a full width at half maximum (FWHM) δE significantly reduced with respect to the occurrence FWHM δE_0_ obtained for F_p_ ≈ 0. Figure 4(g) shows the simulated δE/δE_0_ for 20 (set 1–2) and 40 (set 3) single-electron charge traps and for various charging and discharging rates: l_max_ = 0.3 Hz and f_max_ = 10 Hz for ‘set 1’, l_max_ = 0.1 Hz and f_max_ = 10 Hz for ‘set 2’, and l_max_ = 0.3 Hz and f_max_ = 1 Hz for ‘set 3’ (see Methods). δE/δE_0_ reveals very similar behavior after dropping down for ${{\rm{F}}}_{{\rm{p}}} > {{\rm{F}}}_{{\rm{p}}}^{{\rm{th}}}$ (Fig. 4(g)) for all used modeling parameters.

## Discussion

We observed the modification of the spectral diffusion of QD excitons tuned to resonance with the modes of a photonic nano-cavity. The effect is explained by the Purcell enhancement of the QD-exciton lifetime, which, in turn, reduces the direct charging of the environment by charge released by the QD itself. We note that resonant excitation of QD excitons may avoid charging of the environment by the photoexcited carriers, but does not prevent self-charging by charges confined at the QD via the mechanism invoked in our study. Thus, the reported demonstrations^31,32^ of emission of indistinguishable photons by selected QD-cavity systems using resonant QD pumping are not in contradiction with our observations. Similar to the quenching of emission intermittency in QDs placed in plasmonic nanostructures^20,33^, the use of confined photonic environments for quenching the spectral diffusion of nano-scale light emitters can be useful for the fabrication of novel photon sources.

## Methods

The studied system consists of a single InGaAs/GaAs site-controlled pyramidal QD integrated in an L3 PhC cavity (Fig. 1(a)) fabricated on a 250 nm thick GaAs suspended membrane. The QD is nominally shifted by Δ = 0 nm, Δ = 120 nm or Δ = 180 nm from the center of the L3 PhC cavity (placement precision ~20 nm, confirmed by scanning electron microscopy (SEM)^34^). We used structures with different quantum dot positions in this study because of the limited number of samples of the same design. However, we took care to select structures with both similar QD-CM spectral detuning and similar spectral diffusion behavior. Further details of growth and fabrication can be found in^35–37^.

PhC mode modeling. The near field intensity patterns of the CMs shown in Fig. 1 were calculated in the symmetry plane of the PhC slab using a finite-difference method.

Optical measurements. The QDs were optically excited using a continuous wave Ti:sapphire laser emitting at 730 nm wavelength, with the beam focused to a ~1.5 µm wide spot. Photoluminescence (PL) measurements were carried out with the samples placed in a He-flow optical cryostat using a spectrometer equipped with a charge coupled device (CCD) detector providing a spectral resolution of 80 µeV (reported excitation powers measured before the micro-PL setup). Polarization-resolved spectra were obtained using a λ/2 waveplate and a linear polarizer. Time-resolved PL spectra were measured with 1 s dead time between consecutive acquisitions. Importantly, we avoided any light exposure of the sample from the moment the cooling process started till the PL acquisition.

The reported dynamics of the spectral diffusion were obtained by measuring PL spectra with acquisition time of ~2 s and 1 s dead time between consecutive acquisitions.

Cooling procedure. We avoided any light exposure of the sample before acquisition of the PL spectra in order to achieve reproducible charging conditions. Thus, keeping the sample in complete darkness before the exposure to the laser excitation is crucial for the reported experiments.

Charging/recombination numerical model. In the model, N traps, each either unfilled or filled with a single electron or hole charge, form the charged environment of the QD. We choose an occupation vector $\overrightarrow{{\rm{B}}}={\{{{\rm{B}}}_{{\rm{i}}}\}}_{{\rm{i}}=1}^{{\rm{N}}}$ with B_i_ = 1 (B_i_ = 0) if i^th^ center is charged (not charged). Trap filling with a charge leads to either positive or negative spectral shift^38–43^ of the QD excitonic line (X). Vector $\overrightarrow{{\rm{S}}}={\{{{\rm{\Delta }}E}_{{\rm{i}}}\}}_{{\rm{i}}=1}^{{\rm{N}}}$ describes spectral shifts provided by each of the N centers while it is charged. Spectral shifts are generated randomly from the uniformly distributed numbers in the range [E_min_, E_max_]. The total excitonic energy shift from the unperturbed excitonic energy ${{\rm{E}}}_{{\rm{X}}}^{0}$ is defined as ${\rm{\Delta }}E={\overrightarrow{{\rm{B}}}}^{{\rm{T}}}\ast \overrightarrow{{\rm{S}}}$.

The time evolution of trap occupation is driven by the system of differential equations $\frac{\partial {{\rm{B}}}_{{\rm{i}}}}{\partial {\rm{t}}}={{\rm{f}}}_{{\rm{i}}}(1-{{\rm{B}}}_{{\rm{i}}})-{{\rm{l}}}_{{\rm{i}}}{{\rm{B}}}_{{\rm{i}}}$, i = 1, …, N where f_i_ and l_i_ are charging and discharging rates of i^th^ trap. In our model, charging rate f_i_ is proportional to the probability Π of finding an electron-hole pair in the QD, that is,${f}_{{\rm{i}}}={\rm{\Pi }}\ast {{\rm{F}}}_{{\rm{i}}}$ where F_i_ is the charging rate when the QD is occupied. The steady state solution of the rate equation $\frac{d{\rm{\Pi }}}{{\rm{dt}}}={\rm{P}}(1-{\rm{\Pi }})-{\rm{\gamma }}{\rm{\Pi }}$ provides the QD occupation probability ${\rm{\Pi }}=\frac{1}{1+{\rm{\gamma }}\,/\,{\rm{P}}}$ where γ and P are X recombination and pumping rates, respectively. Charging and discharging rates ${\{{{\rm{F}}}_{{\rm{i}}}\}}_{{\rm{i}}=1}^{{\rm{N}}}$ and ${\{{{\rm{l}}}_{{\rm{i}}}\}}_{{\rm{i}}=1}^{{\rm{N}}}$ are randomly generated from the uniform distribution in the ranges [0, F_max_] and [0, l_max_] tuned for better fitting of the experimental data.

The coupling of the X transition with the CM modifies the QD population depending on the X-CM detuning δ = E − E_CM_, where E_CM_ is the CM energy. The Purcell effect on the X recombination rate reads^28–30^

$${\rm{\gamma }}({\rm{\delta }})=\frac{{{\rm{\gamma }}}_{0}{{\rm{F}}}_{{\rm{P}}}(1+\frac{{{\rm{Q}}}_{{\rm{CM}}}}{{{\rm{Q}}}_{{\rm{X}}}})}{8\frac{{{\rm{\delta }}}^{2}}{{{{\rm{\Delta }}{\rm{\omega }}}_{{\rm{CM}}}}^{2}}+2{(1+\frac{{{\rm{Q}}}_{{\rm{CM}}}}{{{\rm{Q}}}_{{\rm{X}}}})}^{2}}{{\rm{\eta }}}^{2}+{{\rm{\gamma }}}_{{\rm{leak}}}$$

where γ_0_ and γ_leak_ are the X spontaneous emission rates in the bulk (open photonic environment) and in the PhC bandgap, F_P_ is the Purcell factor, ${{\rm{Q}}}_{{\rm{CM}}}=\frac{{{\rm{\omega }}}_{{\rm{CM}}}}{{{\rm{\Delta }}{\rm{\omega }}}_{{\rm{CM}}}}$ and ${{\rm{Q}}}_{{\rm{X}}}=\frac{{{\rm{\omega }}}_{{\rm{X}}}}{{{\rm{\Delta }}{\rm{\omega }}}_{{\rm{X}}}}$ are the CM and the X quality factors defined through the X and the CM angular frequencies ω_CM_ and ω_X_ and their FWHM Δω_CM_ and Δω_X_, and η is a CM-X spatial overlap parameter^44^. For our structure, we chose the Purcell factor F_p_ = 100 and the exciton-CM overlap η = 0.6 providing an emission rate γ enhancement of ~10 times at X-CM resonance (δ = 0) for our QD-PhC cavity parameters: γ_0_ = 0.44 µeV, γ_leak_ = 0.22 µeV, Δω_CM_ = 600 µeV, Q_X_ = 7000 and Q_CM_ = 3000. Thus, we accounted for around 90% linear emission polarization of the X transition observed in resonance with the CM for devices with Δ = 120 nm and Δ = 180 nm.

As initial conditions, we set all traps filled at t = 0, that is, B_i_ = 1 for i = 1, …, N. At each computation interval δt = 0.01 s, charge centers lose or absorb single electrons with probabilities ${{\rm{p}}}_{{\rm{i}}}^{{\rm{loss}}}={{\rm{l}}}_{{\rm{i}}}\ast {\rm{\delta }}t$ and ${{\rm{p}}}_{{\rm{i}}}^{{\rm{abs}}}=\frac{{{\rm{F}}}_{{\rm{i}}}\ast {\rm{\delta }}t\,}{1+{\rm{\gamma }}({\rm{\delta }})\,/\,{\rm{P}}}$, shifting the X transition to a new excitonic energy E(t). This time evolution of the X energy is recorded during t = 10^4^ s providing modeled energy traces of X transition. The occurrence histograms are obtained counting the generated energy values with 50 µeV bin width. The simulated effects did not depend on the particular set of charging and discharging rates F_i_ and l_i_. In the model we introduced both positive and negative shifts by choosing E_min_ < 0 and E_max_ > 0 with in average bigger absolute values for positive shifts, that is, E_max_ > |E_min_| > 0. This is in accordance with the experimentally observed shifts to higher energies of the spectrally diffusing QD transitions relative to the emission energy of the non-diffusing QDs.

Analytical model of excitonic occurrence depletion. To study analytically the observed occurrence depletion, we assumed a linear dependence of the excitonic energy on the total charge Q accommodated in the QD environment: E − E_X_ = const * Q where E_X_ is the excitonic energy at zero charge in QD environment. Assuming continuous charging and discharging of environment with rates α and β, we get the following differential equation driving the time evolution of the charge Q stored in the QD environment:

$$\frac{{\rm{dQ}}}{{\rm{dt}}}=\frac{{\rm{P}}{\rm{\alpha }}}{{\rm{\gamma }}({\rm{\delta }})+{{\rm{\gamma }}}_{{\rm{leak}}}+{\rm{P}}}({{\rm{Q}}}_{{\rm{\max }}}-{\rm{Q}})-{\rm{\beta }}{\rm{Q}}$$

where Q_max_ is the maximum charge that can be stored. Then, the time evolution of excitonic energy reads

$$\frac{{\rm{dE}}}{{\rm{dt}}}=\frac{{\rm{P}}{\rm{\alpha }}({{\rm{E}}}_{{\rm{\max }}}-{\rm{E}})}{\frac{{\rm{C}}1}{{({\rm{E}}-{{\rm{E}}}_{{\rm{CM}}})}^{2}+{\rm{C}}2}+{{\rm{\gamma }}}_{{\rm{leak}}}+{\rm{P}}}-{\rm{\beta }}({\rm{E}}-{{\rm{E}}}_{{\rm{X}}})$$

where E_max_ is the excitonic energy corresponding to the maximum charge accommodated in the environment, ${\rm{C}}1={{\rm{\gamma }}}_{0}{{\rm{F}}}_{{\rm{P}}}(1+\frac{{{\rm{Q}}}_{{\rm{CM}}}}{{{\rm{Q}}}_{{\rm{X}}}})\frac{{{{\rm{\Delta }}{\rm{\omega }}}_{{\rm{CM}}}}^{2}{{\rm{\eta }}}^{2}}{8}$ and ${\rm{C}}2={(1+\frac{{{\rm{Q}}}_{{\rm{CM}}}}{{{\rm{Q}}}_{{\rm{X}}}})}^{2}\frac{{{{\rm{\Delta }}{\rm{\omega }}}_{{\rm{CM}}}}^{2}}{4}\,$where F_P_ is the Purcell factor, ${{\rm{Q}}}_{{\rm{CM}}}=\frac{{{\rm{\omega }}}_{{\rm{CM}}}}{{{\rm{\Delta }}{\rm{\omega }}}_{{\rm{CM}}}}$ and ${{\rm{Q}}}_{{\rm{X}}}=\frac{{{\rm{\omega }}}_{{\rm{X}}}}{{{\rm{\Delta }}{\rm{\omega }}}_{{\rm{X}}}}$ are the CM and the X quality factors defined through the X and the CM angular frequencies ω_CM_ and ω_X_ and their FWHM values Δω_CM_ and Δω_X_, and η is the CM-X spatial overlap. The right part of this equation can be reduced to a single fraction with a numerator being a third degree polynomial of the excitonic energy E providing either one, two or three stationary points E_stat_ of the time-energy curve. Among these points, stable points are provided by the condition $\frac{{{\rm{d}}}^{2}{{\rm{E}}}_{{\rm{stab}}}}{{{\rm{dt}}}^{2}} < 0$.

The datasets generated and analyzed during the current study are available from the corresponding author on reasonable request.
